# Over-expression of microRNA-494 up-regulates hypoxia-inducible factor-1 alpha expression via PI3K/Akt pathway and protects against hypoxia-induced apoptosis

**DOI:** 10.1186/1423-0127-20-100

**Published:** 2013-12-23

**Authors:** Guixiang Sun, Yanni Zhou, Hongsheng Li, Yingjia Guo, Juan Shan, Mengjuan Xia, Youping Li, Shengfu Li, Dan Long, Li Feng

**Affiliations:** 1 Key Laboratory of Transplant Engineering and Immunology of Health Ministry of China, West China Hospital, Sichuan University, Chengdu 610041, Sichuan, Province, PR China; 2 Regenerative Medicine Research Center, West China Hospital, Sichuan University, Chengdu, Sichuan, Province, PR China; 3 Chinese Cochrane Centre, Chinese Evidence-Based Medicine Centre, West China Hospital, Sichuan University, Chengdu, Sichuan, Province, PR China

**Keywords:** MicroRNA-494, Hypoxia-inducible factor-1 alpha, PI3K/Akt, Apoptosis, L02 cells

## Abstract

**Background:**

Hypoxia-inducible factor-1 alpha (HIF-1α) is one of the key regulators of hypoxia/ischemia. MicroRNA-494 (miR-494) had cardioprotective effects against ischemia/reperfusion (I/R)-induced injury, but its functional relationship with HIF-1α was unknown. This study was undertaken to determine if miR-494 was involved in the induction of HIF-1α.

**Results:**

Quantitative RT-PCR showed that miR-494 was up-regulated to peak after 4 hours of hypoxia in human liver cell line L02. To investigate the role of miR-494, cells were transfected with miR-494 mimic or miR-negative control, followed by incubation under normoxia or hypoxia. Our results indicated that overexpression of miR-494 significantly induced the expression of p-Akt, HIF-1α and HO-1 determined by qRT-PCR and western blot under normoxia and hypoxia, compared to negative control (*p* < 0.05). While LY294002 treatment markedly abolished miR-494-inducing Akt activation, HIF-1α and HO-1 increase under both normoxic and hypoxic conditions (*p* < 0.05). Moreover, apoptosis detection using Annexin V indicated that overexpression of miR-494 significantly decreased hypoxia-induced apoptosis in L02 cells, compared to control (*p* < 0.05). MiR-494 overexpression also decreased caspase-3/7 activity by 1.27-fold under hypoxia in L02 cells.

**Conclusions:**

Overexpression of miR-494 upregulated HIF-1α expression through activating PI3K/Akt pathway under both normoxia and hypoxia, and had protective effects against hypoxia-induced apoptosis in L02 cells. Thus, these findings suggested that miR-494 might be a target of therapy for hepatic hypoxia/ischemia injury.

## Background

MicroRNAs (miRNAs) are small non-coding RNAs with the length of 21- to 25-nucleotides that posttranscriptionally regulate the expression of target genes, and play important roles in various biological processes, including development, differentiation, proliferation, and apoptosis [1,2]. Several studies have suggested that alterations of their expression may paly a role in the regulation of the cellular response to hypoxia [3-6].

Hypoxia availability affects cells and tissues during normal embryonic development and pathological conditions such as myocardial infarction, inflammation and tumorigenesis. Hypoxia inducible factor-1 (HIF-1) is recognized as the master transcription factor consisting of a constitutively expressed HIF-1β subunit and an oxygen-regulated HIF-1α subunit in response to hypoxia [7]. In normoxia, HIF-1α is maintained at lower level by proteasomal degradation [8]. During hypoxia the degradation of HIF-1α is inhibited, and then HIF-1α heterodimerizes with HIF-1β and translocates to the nucleus [7]. HIF-1α/β dimer binds to hypoxia response elements (HREs) and activates target genes transcription, including heme oxygenase-1 (HO-1), erythropoietin (EPO), vascular endothelial growth factor (VEGF), and various glycolytic enzymes that contribute to adaptation to hypoxia and/or ischemia [9]. Therefore HIF-1α plays a key role in hypoxic/ischemic response.

Recent studies indicate that miRNAs play important roles in hypoxia/ischemia [3,10-16]. MiR-494 has been reported to be significantly increased in ex vivo ischemia/reperfusion (I/R) mouse hearts [16]. Moreover, miR-494 has cardioprotective effects against ischemia/reperfusion-induced injury by targeting both proapoptotic proteins (PTEN, ROCK1, CaMKIIδ) and antiapoptotic proteins (FGFR2 and LIF) to active the Akt-mitochondrial signaling pathway [16]. Obviously, HIF-1α plays an important role in hypoxia and/or ischemia conditions. Studies have shown that Akt can augment HIF-1α expression by increasing its translation under both normoxic and hypoxic conditions [17-19]. However, the potential link between miR-494 and HIF-1α is unknown. We hypothesize that miR-494 may have a role in influencing HIF-1α expression and contribute to the cellular response to hypoxia. Simultaneously, almost all previous studies about miR-494 were implemented in tumour cells or myocardial cell. The role of miR-494 in liver cell was unclear. Therefore, the present study was undertaken to investigate the influence of miR-494 on HIF-1α expression and its relative mechanism in human hepatic cell line L02. We also investigated the function of miR-494 in response to hypoxia-induced apoptosis. Our results showed that miR-494 were upregulated up to peak after 4 h of hypoxia in the L02 human hepatic cell line. Furthermore, we found that overexpression of miR-494 increased the of expression HIF-1α through activating the PI3K/Akt signaling pathway and protected against hypoxia-induced apoptosis in the immortalized hepatocyte cell line L02.

## Methods

### Cell culture

The L02 human hepatic cell line purchased from China Center for Type Culture Collection (Wuhan, China) was cultured in RPMI 1640 medium (Gibco) supplemented with 10% fetal bovine serum (FBS). Cells were grown under normoxic (21% O_2_) or hypoxic (1% O_2_) conditions at 37°C/5% CO_2_. Specially, medium was replaced with Dulbecco’s modified Eagle’s medium (DMEM; Gibco) without serum and glucose during hypoxia. To block PI3K/Akt signaling pathway, LY294002 (PI3K inhibitor, 30 μmol/L; Sigma-Aldrich) was added to the culture medium.

### MiRNA and cell transfection

MiR-494 mimic and the negative control were obtained from RiboBio (Guangzhou, China). The miR-494 overexpression study was performed using miR-494 mimic (200 nM) and its negative control (200 nM). Cells were cultured to 30-50% confluence, and transfected with miR-494 mimic and negative control using Lipofectamine 2000 (Invitrogen) in serum-free Opti-MEM medium (Gibco) according to the manufacturer’s instruction. Cells were cultured in fresh medium containing 10% FBS after transfection. Transfected cells were cultured for 48 hours under normoxia (21%O_2_, 5%CO_2_ in a 37°C incubator), or grown under normoxia for 16 hours prior to exposure to hypoxia (1%O_2_, 5%CO_2_ in a 37°C incubator) for 8 hours. After hypoxia, apoptosis was analyzed using Annexin V-FITC/PI binding staining and caspase-3/7 activity were measured by Cytomics™ FC500 flow cytometer (Beckman Coulter, USA). Total RNAs and protein were prepared for real-time reverse transcription-polymerase chain reaction (RT-PCR) and western blot analysis.

### RNA extraction and real-time RT-PCR

Total RNA was extracted from cultured cells using Trizol (Invitrogen, Carlsbad, CA). The levels of mRNAs or miRNAs were measured by real-time quantitative RT-PCR (qRT-PCR) using Bio-Rad IQ5 system. For mRNA detection, reverse transcription was performed with PrimeScript™ RT reagent kit (TaKaRa, Dalian, China) according to the manufacturer’s instructions, and real-time RT-PCR was carried out using SsoFast™ EvaGreen Supermix kit (Bio-Rad) with Bio-Rad IQ5 real-time PCR system. The real-time PCR reaction contained: 10 μL of SsoFast EvaGreen supermix, 1 μL of sense primer, 1 μL of anti-sense primer, 2 μL of cDNA template, and 6 μL of H_2_O. The program of two step real time RT-PCR was 95°C for 30 seconds, followed by 40 cycles of 95°C for 5 seconds, and 60°C for 10 seconds. The relative expression level of mRNAs was normalized to that of internal control β-actin by using the 2^-ΔΔCt^ cycle threshold method. Primer sequences were as follows:

HIF-1α sense primer, 5′-CAAGAACCTACTGCTAATGC-3′; HIF-1α anti-sense primer, 5′-TTATGTATGTGGGTAGGAGATG-3′; VEGF sense primer, 5′-ACAGGGAAGAGGAGGAGATG-3′; VEGF anti-sense primer, 5′-GCTGGGTTTGTCGGTGTTC-3′; HO-1 sense primer, 5′-GCCAGCAACAAAGTGCAAGA-3′; HO-1 anti-sense primer, 5′-AAGGACCCATCGGAGAAGC-3′; β-actin sense primer, 5′-AAGATCATTGCTCCTCCTG-3′; β-actin anti-sense primer, 5′-CGTCATACTCCTGCTTGCTG-3′.

To detect the level of mature miR-494, the complementary DNA (cDNA) was synthesized using PrimeScript™ RT reagent kit (TaKaRa, Dalian, China) and miRNA-specific stem-loop RT primers (RiboBio, Guangzhou, China). The 10 μL of reaction contained: 2 μL of 5× RT buffer, 0.5 μL of PrimeScript™ RT Enzyme Mix, 1 μL of miR-494 RT primer, 1 μL of total RNA (<500 ng), and 5.5 μL of H_2_O. The incubation condition was 37°C for 15 minutes, followed by 85°C for 5 seconds. Then qRT-PCR was performed with SsoFast™ EvaGreen Supermix kit (Bio-Rad) and Bio-Rad IQ5 real-time PCR system. The reaction contained: 10 μL of SsoFast EvaGreen supermix, 1.5 μL of forward primer, 1.5 μL of reverse primer, 2 μL of cDNA template, and 5 μL of H_2_O. The program was the same as that described above. Forward and reverse primers were designed from RiboBio (Guangzhou, China). U6 small nuclear RNA was used as an internal control.

### Protein extraction and western blot analysis

Cells were washed twice quickly with ice-cold phosphate buffered saline (PBS) after either hypoxic or normoxic incubation, solubilized in 1× lysis buffer [50 mmol/L Tris (pH 6.8), 2%SDS, 10% glycerol] with protease inhibitors (Complete, EDTA-free tablets, Roche) and phosphatase inhibitors (Roche) on ice. Cell lysates were sonicated in an Ultrasonic Dismemberator on ice, followed by boiling for 5 minutes and centrifuging at 12000 *g* for 10 minutes at 4°C and the supernatants were retained. Protein concentration was determined by a BCA Protein Assay kit (Beyotime, Shanghai, China).

For western blot, equal amounts of total protein in special condition (40 μg for hypoxic condition, 80 μg for normoxic condition) were loaded for electrophpresis in sodium dodecyl sulfate-polyacrylamide (SDS) gels and then transferred to polyvinylidene fluoride microporous membranes (PVDF; Bio-Rad). After blocking [5% non-fat dry milk in Tris-buffer saline (TBS) and 0.1% Tween-20] for 1 hour at room temperature, the membranes were incubated with the primary antibodies overnight at 4°C. The following antibodies were used in this study: monoclonal antibody HIF-1α (1:1000; Abcam), phospho-Akt (Ser308, 1:1000) and Akt (1:1000; Cell Signaling Technology), monoclonal antibody PTEN (1:1000; R&D), monoclonal antibody HO-1(1:1000; EPITOMICS, USA) and monoclonal antibody β-Tubulin (1:1000; CWBIO, China). The membranes were washed three times with 1× TBST, followed by incubation with HRP-conjugated anti-rabbit or anti-mouse immunoglobulin G secondary antibodies (1:2000; Cell Signaling Technology) for 1 hour at 37°C. The membranes were detected with enhanced chemiluminescence plus reagents (Millipore) after washing. The band images were densitometrically analyzed using Quantity one software (Bio-Rid). β-Tubulin was used as an internal control.

### Annexin V and phosphatidylinositol (PI) binding staining

The assay of Annexin V and PI binding staining was performed with an Annexin V-FITC Apoptosis Detection Kit according to the manufacturer’s instructions (Keygen Biotech, Nanjing, China). In short, cells after hypoxia were digested with 0.25% trypsin without EDTA, and then washed twice with cold PBS, centrifuged at 3000 rpm for 5 minutes. Cells were resuspended in 500 μL of 1× binding buffer at a concentration of 5 × 10^5^ cells/mL, 5 μL Annexin V-FITC and 5 μL PI were added. Cells were gently mixed and incubated for 10 minutes at 37°C in the dark. Transfer 400 μL of cell suspension to flow tubes. Stained cells were analyzed by Cytomics™ FC500 flow cytometer (Beckman Coulter, USA).

### Caspase-3/7 activity assay

After hypoxia, caspase activity was measured with a Vybrant FAM Caspase-3 and Caspase-7 Assay Kit according to the manufacturer’s instructions (Invitrogen). Briefly, cells after hypoxia were harvested and resuspended in culture media at a concentration of 1 × 10^6^ cells/mL. 300 μL of cell suspension were transferred to each centrifugal tube, 10 μL of 30× FLICA working solution were added. Cells were gently mixed and incubated for 60 minutes at 37°C/5%CO_2_ in the dark, followed by twice washing with 1× wash buffer, pelleted the cells by centrifugation of 3000 rpm for 5 minutes. Cells were resuspended in 400 μL of 1× wash buffer, and then 2 μL of PI were added. Cell suspension was incubated for 5 minutes on ice in the dark. 400 μL of stained cells were transferred to flow tubes and analyzed on the flow cytometer.

### Statistical analysis

All data were expressed as mean ± SD. Statistical analysis was performed using double-sided Student’s *t* test or one-way ANOVA by SPSS 13.0. *P* value less than 0.05 was considered statistically significant difference.

## Results

### Hypoxia-induced changes in miRNA-494 expression in human hepatic cell line L02

**Figure 1:**
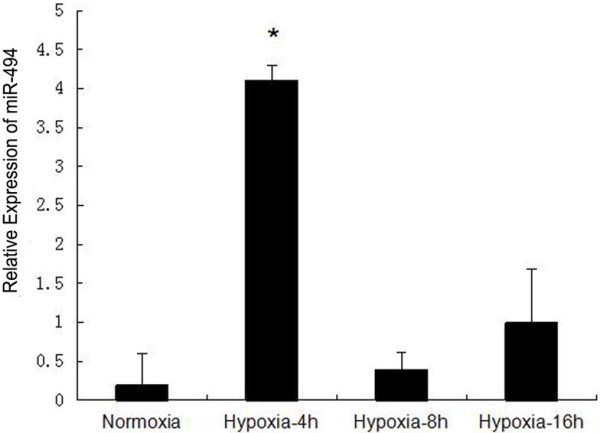
**Hypoxia induced upregulation of miR-494 in L02 cells.** Cells were incubated under hypoxia (1%O_2_, 5%CO_2_ in a 37°C incubator) for 4 hours, 8 hours, and 16 hours, followed by analysis for miR-494 expression by real-time qRT-PCR. U6 small nuclear RNA was used as an internal control. The data were presented as the means ± SD. Columns, mean of three independent experiments; bars, SD; *p < 0.05 compared to other three groups.

In the present study, we wonder about the hypoxia-induced changes in miRNA-494 expression in L02 cells. Our results indicated that miR-494 levels were significantly upregulated after hypoxia for 4 hours, followed by decrease under further hypoxia (Figure 1). The changes were similar to that in ex vivo ischemic mouse hearts [16]. These findings indicated that alteration of miR-494 was dependent on the physiological/pathological conditions. We hypothesized that upregulation of miR-494 might represent an adaptive response to early hypoxia challenge.

### MiR-494 overexpression increased HIF-1α and HO-1 expression under normoxia and hypoxia

**Figure 2:**
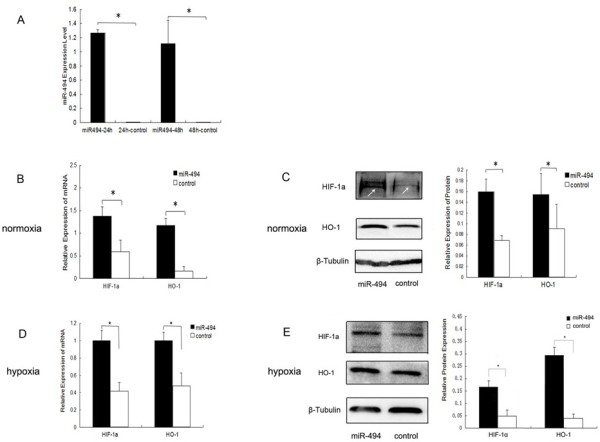
**Overexpression of miR-494 increased HIF-1α and HO-1 expression under both normoxia and hypoxia. (A)** Expression of miR-494 in transfected L02 cells. L02 cells were transfected with either miR-494 mimic or miR-negative control at 200 nM under normoxia. MiR-494 levels were analyzed by real-time qRT-PCR at 24 hours for transfection and 48 hours for transfection, respectively. **(B-C)** Expression of HIF-1α and HO-1 in transfected L02 cells under normoxia. L02 cells were transfected with either miR-494 mimic or miR-negative control at 200 nM under normoxia. After transfection for 48 hours, total RNAs and proteins were extracted and subjected to real-time qRT-PCR **(B)** or western blot assay (**C**. Left: Western blot analysis for HIF-1α, HO-1 and β-Tubulin. The sample loading of protein for normoxic condition was 80 μg. The signal indicated by white arrow is HIF-1α. Right: Densitometric analysis using Quantity one software in left). **(D-E)** Expression of HIF-1αand HO-1 in transfected L02 cells under hypoxia. After transfection for 16 hours, cells were exposed to hypoxia (1%O_2_, 5%CO_2_ in a 37°C incubator) for 8 hours. Total RNAs and proteins were extracted, and then real-time qRT-PCR **(D)** and western blot (**E**. Left: Western blot analysis for HIF-1α, HO-1 and β-Tubulin. The sample loading of protein for hypoxic condition.was 40 μg. Right: Densitometric analysis using Quantity one software in left) were done. Control indicated miR-negative control. The quantitative data for western blot were normalized by the level of β-Tubulin expression. The data were presented as the means ± SD. Columns, mean of three independent experiments; bars, SD; *p < 0.05.

To detect the effect of miR-494 overexpression on HIF-1α expression, L02 cells were transfected with miR-494 mimic or miR-negative control via Lipo2000. Comparing with the negative control group, the expression of miR-494 in mimic transfection group was significantly increased after transfection for 24 hours and 48 hours, respectively (Figure 2A), indicating that miR-494 overexpression system in L02 cells was successful in technology.

Functionally, we found that overexpression of miR-494 significantly increased mRNA and protein levels of HIF-1α under normoxia, resulted in the subsequence expression of downstream target gene HO-1 (*p* < 0.05) (Figure 2B, C). To assess the effect of miR-494 on HIF-1α under hypoxia, transfected cells were exposed to hypoxia (1%O_2_, 5%CO_2_ in a 37°C incubator) for 8 hours. Our results showed that overexpression of miR-494 also significantly increased mRNA and protein levels of HIF-1α and HO-1 (p < 0.05) (Figure 2D, E). These results suggested that overexpression of miR-494 increased HIF-1α and HO-1 expression levels under both normoxic and hypoxic conditions in L02 cells.

### MiR-494 increased HIF-1α expression through PI3K/Akt pathway

**Figure 3:**
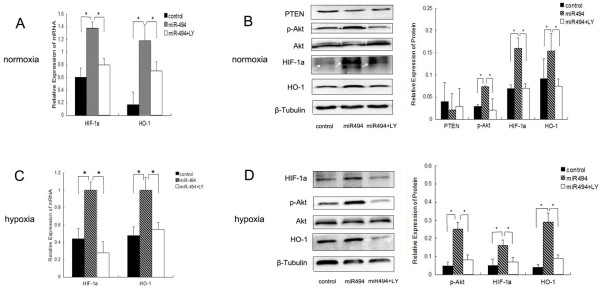
**MiR-494 induced HIF-1α and HO-1 expression by activating PI3K/Akt pathway. (A)** Cells were transfected with miR-494 mimic or miR-negative control under normoxia as described above. After transfection, cells were treated with or without 30 μM of LY294002 (PI3K inhibitor, block the PI3K/Akt pathway) for 8 hours. mRNA levels of HIF-1α and HO-1 were analyzed by real-time RT-PCR after transfection for 48 hours. **(B)** The expression of PTEN, p-Akt, Akt, HIF-1α, HO-1 and β-Tubulin were analyzed by western blot after 48 hours for transfection. Left: Western blot analysis for proteins. The signal indicated by white arrow is HIF-1α. Right: Densitometric analysis of the western blot in left. **(C-D)** Transfected cells were continuously treated with or without 30 μM of LY294002 for 16 hours prior to hypoxia (1% O_2_, 5% CO_2_ in a 37°C incubator). Eight hours later, cells were harvested for RNA and protein, real-time RT-PCR **(C)** and western blot (**D**. Left: Western blot analysis for proteins. Right: Densitometric analysis using Quantity one software in left) were done. Control indicates miR-negative control. MiR-494 + LY indicates treatment with LY294002 in transfected cells. Data were normalized by the level of β-Tubulin expression except p-Akt in panel **B** and panel **D**. The data of p-Akt was normalized by the level of Akt expression. The data were presented as the means ± SD. Columns, mean of three independent experiments; bars, SD; *p < 0.05.

Several studies revealed that miR-494 could target PTEN, leading to activate PI3K/Akt pathway which could augment HIF-1α expression [17,20-23]. To confirm whether miR-494 increased HIF-1α expression through PTEN/PI3K/Akt pathway in L02 cells, we detected proteins expression of PTEN, p-Akt, HIF-1α and its target gene HO-1. We found that mRNA levels of HIF-1α and HO-1 were increased by miR-494 (Figure 3A). Overexpression of miR-494 induced Akt activation and significantly increased HIF-1α and HO-1 expression under normoxia, compared to negative control (*p* < 0.05). While the significant decrease of PTEN was not observed (Figure 3B). Similarly, overexpression of miR-494 also increased mRNA levels of HIF-1α and HO-1 under hypoxia (Figure 3C), and upregulated proteins expression of p-Akt, HIF-1α and HO-1 in L02 cells (*p* < 0.05) (Figure 3D).

To further establish the axis of miRNA-494/p-Akt/HIF-1α, cells were transfected with miR-494 mimic and treated with LY294002 (PI3K inhibitor, block the PI3K/Akt pathway) at 30 μM. LY294002 treatment inhibited miR-494-inducing HIF-1α and HO-1 mRNA levels (Figure 3A, C), and abolished miR-494-inducing Akt activation leading to subsequent decrease of HIF-1α and HO-1 protein levels under both normoxic and hypoxic conditions (*p* < 0.05) (Figure 3B, D). These results suggested that overexpression of miR-494 could augment HIF-1α expression through Akt activation in L02 cells. However, more studies are needed to determine whether miR-494 activate the Akt pathway by targeting PTEN in L02 cells.

### Overexpression of miR-494 protected L02 cells against hypoxia-induced apoptosis

**Figure 4:**
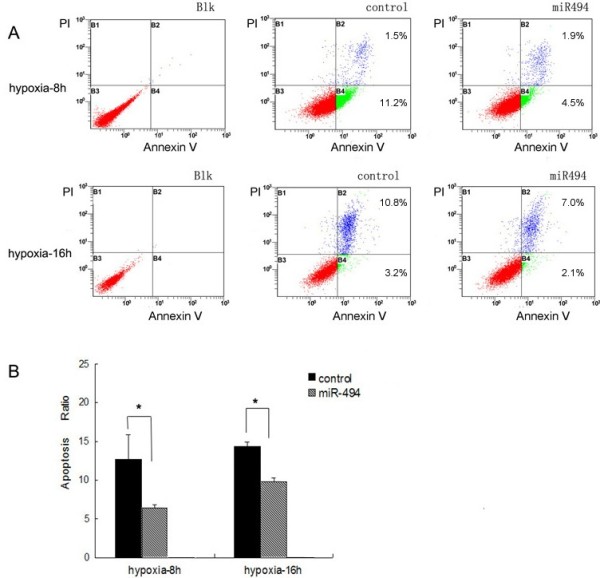
**Effect of miR-494 on hypoxia-induced apoptosis was determined with Annexin V-FITC/PI binding staining by flow cytometry. (A)** Flow cytometry results with Annexin V-TITC/PI staining. Cells were transfected with miR-494 mimic or miR-negative control as described above. After hypoxia for 8 hours (Top) or 16 hours (Bottom), cells were harvested and then apoptosis was analyzed with an Annexin V-FITC Apoptosis Detection Kit by flow cytometry. Cells were classified as healthy cells (Annexin V^−^, PI^−^), early apoptotic cells (Annexin V^+^, PI^−^), late apoptotic cells (Annexin V^+^, PI^+^), and damaged cells (Annexin V^−^, PI^+^). **(B)** The ratio of apoptosis among different experimental groups. Apoptosis ratio was early apoptosis percentage plus late apoptosis percentage. Control indicated miR-negative control. The data were presented as the means ± SD. Columns, mean of three independent experiments; bars, SD; *p < 0.05.

To determine the effect of miR-494 on hypoxia-induced apoptosis in L02 cells, transfected cells incubated under hypoxia were stained with Annexin V-FITC/PI and detected by flow cytometry (Figure 4). We found that most of apoptotic cells were at an early apoptotic state after hypoxia for 8 h, but at a late apoptotic state after further hypoxia for 16 h (Figure 4A). The apoptosis ratio in miR-494 mimic group was significantly decreased comparing with control group both under hypoxia for 8 h and 16 h (*p* < 0.05) (Figure 4B).

**Figure 5:**
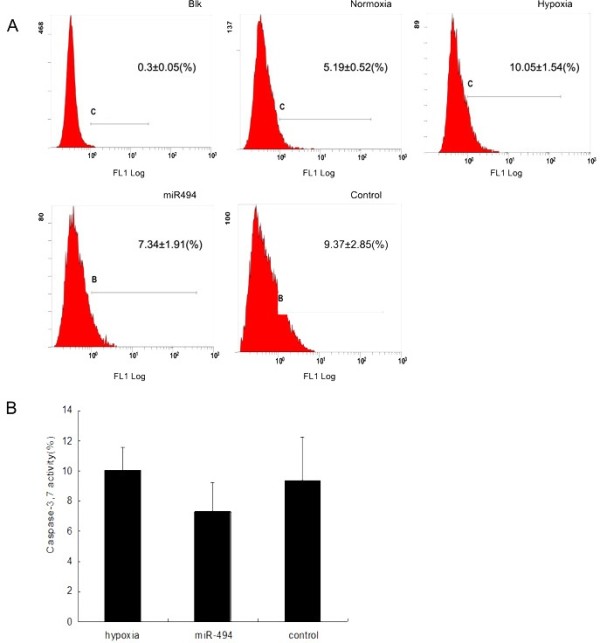
**Caspase-3 and caspase-7 activity in transfected cells under hypoxia.** Transfected cells were incubated under hypoxia for 8 hours. Cells were harvested and Caspase-3/7 activity were determined using the Caspase-3 and Caspase-7 Assay Kit by flow cytometry. **(A)** Flow cytometry results for caspase-3/7 activity. **(B)** Histogram of the percentage of caspase-3/7 activity among different groups. Control indicated miR-negative control. The data were presented as the means ± SD. Columns, mean of three independent experiments; bars, SD.

In addition, hypoxia-induced caspase-3/7 activity in L02 cells were assessed using a Vybrant FAM Caspase-3 and Caspase-7 Assay Kit for flow cytometry (Figure 5). After 8 hours of incubation in hypoxia, caspase-3/7 activity in miR-494mimic-transfected L02 cells decreased by 1.27-fold compared with negative control. However, there were no statistical differences in the caspase-3/7 activity between groups (Figure 5B).

Together, these findings provided evidence that overexpression of miR-494 might protect L02 cells against hypoxia-induced apoptosis. While further study is needed to confirm this conclusion.

## Discussion

Previous studies have demonstrated that miR-494 could target both proapoptotic proteins and antiapoptotic proteins to active the Akt-mitochondrial signaling pathway, leading to cardioprotective effects against ischemia/reperfusion-induced injury [16]. HIF-1α plays a key role in several hypoxia-related physiologic and pathophysiologic responses, involving embryogenesis, ischemic injury and tumorigenesis [24]. However, the relationship between miR-494 and HIF-1α has not been explored. Our study is first to reveal the role of overexpression of miR-494 in regulating HIF-1α expression in L02 cells. In this study, we have shown that overexpression of miR-494 in L02 cells increased the expression of HIF-1α and its downstream gene HO-1 by activating the PI3K/Akt pathway. We found that overexpression of miR-494 had protective effects against hypoxia-induced apoptosis in L02 cells.

The role of HIF-1α as a nuclear factor has been studied extensively [25-27]. In normoxia, HIF-1α is hydroxylated by proline hydroxylase (PHD), and then recognized by the von Hippel-Lindau protein (vHL) resulting in proteosomal degradation [25]. This process is inhibited during hypoxia. HIF-1α can move into the nucleus to form an active complex with HIF-1β and CBP/p300, resulting in transcription of target genes [7]. Several regulators and mechanisms regulate the stability and activity of HIF-1α protein. Recent studies indicate that miRNAs play important roles in hypoxic adaptation [2]. Many miRNAs that regulate the expression of HIF-1α directly or indirectly are detected, such as miR-210, miR-519c, miR-20a and miR-21 [3,11,15,19,28]. One specific microRNA, miR-494 has been studied in cancer research and got more and more attention [20,29-31]. While several miRs profiling studies revealed that miR-494 was downregulated in animal ischemic/hypertrophic hearts [32,33], Xiaohong Wang et al. reported that miR-494 levels were increased in ex vivo I/R mouse hearts [16]. In present study, we found that miR-494 was upregulated in L02 cells during hypoxia (Figure 1), which might represent an adaptive response to hypoxia challenge. Though miR-494 was significantly increased during hypoxia for 4 hours in L02 cells. Transfected cells were exposed to hypoxia for 8 hours in our following study, because there was a more obvious difference of HIF-1α expression after 8 hours of hypoxia between miR-494 mimic group and miR-negative control group (data not shown).

We used the microRNA target prediction websites TargetScan and mcroRNA.org to predict the relationship between miR-494 and HIF-1α. We found that there were no targets for miR-494 in 3’ UTR of HIF-1α. Our results also showed that overexpression of miR-494 increased the expression of HIF-1α and its downstream gene HO-1 under normoxia and hypoxia in L02 cells (Figure 2). It suggested that miR-494 induced HIF-1α expression through some other pathways, not direct regulation.

Furthermore, we investigated the mechanism of miR-494 regulating HIF-1α in L02 cells. A series of studies have revealed that miR-494 played an important role in tumor [23,34,35]. miR-494 targeted PTEN resulting in the subsequent activation of the Akt pathway involved in various pathophysiologic processes, including cell apoptosis, survival, tumor metastasis, and angiogenesis [20,22,23]. It has been reported that miR-494 had cardioprotective effects against ischemia/reperfusion-induced injury through Akt activation [16]. In our study, western blot analysis results showed that overexpression of miR-494 could markedly enhance Akt phosphorylation leading to the subsequent upregulation of HIF-1α and HO-1under normoxia and hypoxia, compared to control group (Figure 3). Treatment of the L02 cells with PI3K inhibitor LY294002 inhibited miR-494-inducing HIF-1α and HO-1 expression (Figure 3). Taken together, we supposed that miR-494 induced HIF-1α expression dependent on Akt activation. Of course, we could not exclude that other signaling molecules also contributed in miR-494-inducing HIF-1α expression. Actually, our results were similar with the mechanism of miR-21-mediated HIF-1α expression that overexpression of miR-21 increased HIF-1α and VEGF expression by activating AKT and ERK pathway [19]. While the direct target genes of miR-494 should be demonstrated in our future study.

To further study the biological function of miR-494 in hypoxia, cell apoptosis was detected by Annexin V-FITC/PI staining and caspase-3/7 activity were analyzed by flow cytometry. Annexin V-FITC could recognize the cell membrane exposure of phosphatidylserine normally restricted to the inner cell membrane in the early apoptotic stage [36]. The late apoptotic stage was assessed by measuring the DNA labeling with the PI. Our results showed that overexpression of miR-494 decreased apoptosis ratio under hypoxia comparing with negative control (Figure 4). Simultaneously, caspase-3/7 are key executioners of apoptosis, and the activities of them can reflect levels of cell apoptosis, especially for an early apoptotic state [37]. We found that caspase-3/7 activity were decreased by 1.27-fold in miR-494mimic-transfected cells (Figure 5). Unfortunately, there were no statistical significance differences (*p* >0.05). These data suggested that miR-494 had protective effects against hypoxia-induced apoptosis in L02 cells. But more experiments were needed to confirm the conclusion.

## Conclusions

In conclusion, our investigations demonstrated that overexpression of miR-494 could augment HIF-1α expression through Akt activation in L02 cells for the first time. During hypoxia, overpression of miR-494 protected L02 cells against hypoxia-induced apoptosis. Our data may be useful for further relative researches and contribute to development of a new therapy for hepatic hypoxia/ischemia injury.

## Competing interests

The authors declare that they have no competing interests.

## Authors’ contributions

GXS was responsible for the writing of the manuscript. YNZ and HSL carried out cell transfection and real-time RT-PCR experiments. YJG and JS carried out the apoptosis detection and analysis. SFL, DL and MXJ were involved in the cell culture and Western blot experiment. YPL performed the statistical analysis. LF conceived of the study, and participated in its design and coordination and helped to draft the manuscript. All authors read and approved the final manuscript.
